# Heterozygous pathogenic variants in *CWF19L1* in a Chinese family with spinocerebellar ataxia, autosomal recessive 17

**DOI:** 10.1002/jcla.24767

**Published:** 2022-11-10

**Authors:** Miaohua Ruan, Hongwei Wang, Mianmian Zhu, Rongyue Sun, Jiamin Shi, Qiu Wang, Yuan Chen, Yihong Wang, Dan Wang

**Affiliations:** ^1^ Department of Pediatrics The First Affiliated Hospital of Wenzhou Medical University Wenzhou China; ^2^ Key Laboratory of Diagnosis and Treatment of Severe Hepato‐Pancreatic Diseases The First Affiliated Hospital of Wenzhou Medical University Wenzhou China

**Keywords:** autosomal recessive cerebellar ataxia, compound heterozygous variants, *CWF19L1*, novel variant

## Abstract

**Background:**

*CWF19L1 i*s responsible for spinocerebellar ataxia, autosomal recessive 17, which presents with cerebellar ataxia, and atrophy. Here, we report novel compound heterozygous variants of *CWF19L1* in a Chinese family with progressive ataxia and mental retardation of unknown etiology by analyzing clinical characteristics and genetic variations.

**Methods:**

Clinical profiles and genomic DNA extracts of family members were collected. Whole‐exome and Sanger sequencing were performed to detect associated genetic variants. Pathogenicity prediction and conservation analysis of the identified variants were performed using bioinformatics tools.

**Results:**

We identified heterozygous variants at the invariant +2 position (c.1555_c.1557delGAG in exon 14 and c.1070G > T in exon 11) of the *CWF19L1* gene. Two novel heterozygous variants of the *CWF19L1* gene were identified in the *CWF19L1* gene associated with autosomal recessive cerebellar ataxia.

**Conclusion:**

Our results suggest that *CWF19L1* variants may be a novel cause of recessive ataxia with developmental delay. Whole‐exome sequencing is an efficient tool for screening variants associated with the disease. This case report may help diagnose and identify the causes of other ataxias, leading to novel therapies, especially in China. This finding enriches the variant spectrum of the *CWF19L1* gene and lays the foundation for future studies on the correlation between genotype and phenotype.

## Graphical abstract

In this article, we report novel compound heterozygous variants of *CWF19L1* in a Chinese family with progressive ataxia and mental retardation of unknown etiology by analyzing clinical characteristics and genetic variation. Whole‐exome sequencing and Sanger sequencing were used to detect associated genetic variants. We identified heterozygous variants at the invariant +2 position (c.1555_c.1557delGAG) in exon 14 and (c.1070G > T) in exon 11 of the *CWF19L1* gene. Pathogenicity prediction and conservation analysis of the identified variants were performed with bioinformatics tools. This finding enriches the variant spectrum of the *CWF19L1* gene and lays the foundation for future studies on the correlation between genotype and phenotype.

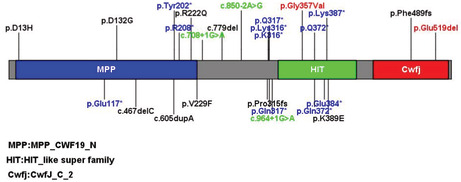

## 1 INTRODUCTION

Autosomal recessive cerebellar ataxia (ARCA) is a rare neurological disorder that affects both the peripheral and central nervous systems and occurs primarily in children.^1^, ^2^, ^3^ The incidence of ARCA has been reported to be 0–7.2 per 100,000 children.^4^ Previous studies have described that patients have varied clinical manifestations, ranging from progressive cerebellar syndromes, as a key feature, to spinocerebellar symptoms with spasticity, ophthalmoplegia, involuntary movements, seizures, cognitive impairment, and peripheral neuropathy.^1^, ^2^, ^3^, ^4^, ^5^ Over the past two decades, more than 50 genes have been reported to cause ARCA, accounting for only 40% of cases.^6^, ^7^ One of these genes is *CWF19L1* on chromosome 10q24,^8^ which was recently identified and characterized to cause ARCA.^9^, ^10^, ^11^

**FIGURE 1:**
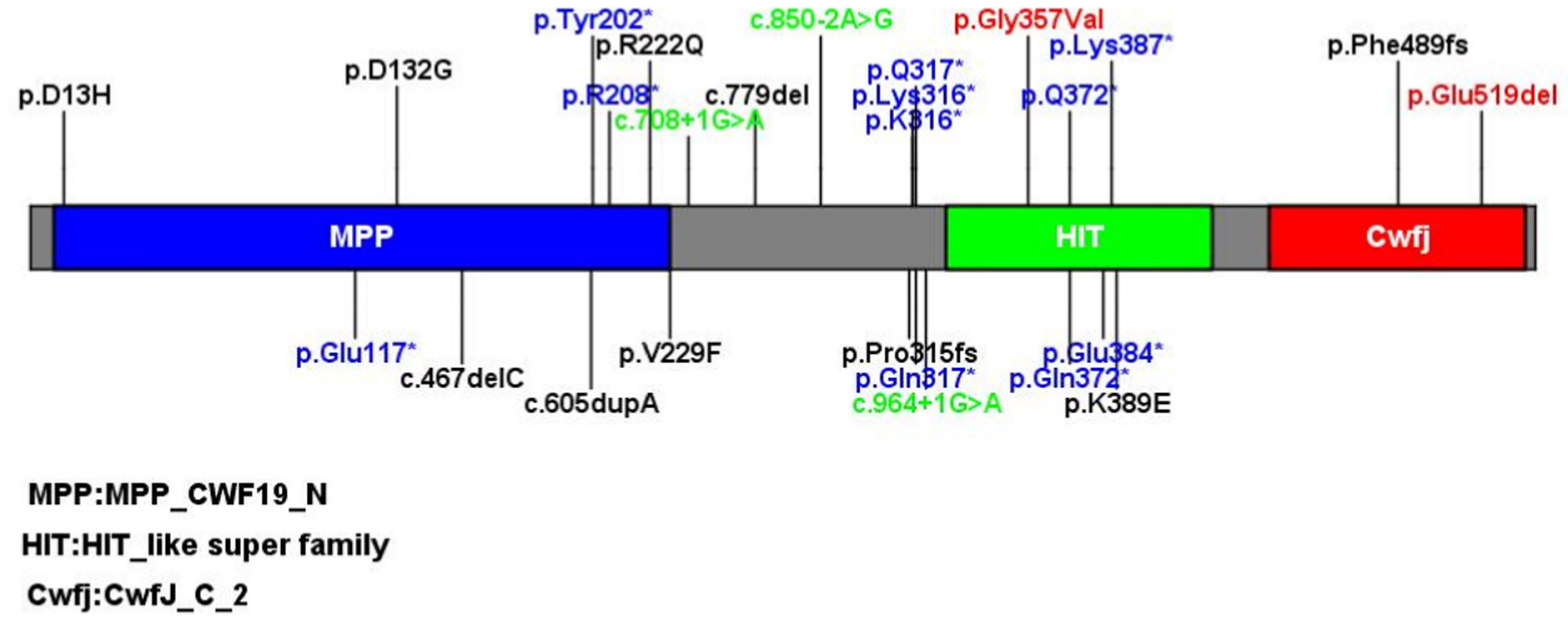
Schematic presentation of linear C19L1 protein with all variants. Red font indicates reported variants in the case report.

The C19L1 protein encoded by the *CWF19L1* gene contains 538 amino acids, including a metallophosphatase (MMP) domain, a histidine triad motif (HIT) domain, and a C‐terminal Cwf‐J‐C domain^12^, ^13^ (Figure 1). Only 26 *CWF19L1* variants have been reported globally in the Human Gene Mutation Database and ClinVar database (Figure 1). Consistent with the studies reporting that variants in *CWF19L1* may cause ARCA,^8^, ^9^, ^10^, ^11^ we postulated that novel variants in *CWF19L1* in our study may be related to ARCA in this family. However, neither such a variant has been reported in Chinese patients, nor has the same variant been previously described in the literature. We herein present a case of a boy diagnosed with two previously unreported variants in the *CWF19L1* gene based on genetic analysis and clinical characteristics.

## 2 MATERIALS AND METHODS

### 2.1 Participants

The proband and his parents were enrolled at the First Affiliated Hospital of Wenzhou Medical University. Written consent was obtained from the parents of the patients before starting the study. All study protocols were reviewed by the Ethics Committee of the First Affiliated Hospital of Wenzhou Medical University (2016–187).

### 2.2 Whole‐exome and Sanger sequencing

Whole‐exome sequencing was performed on the proband. Genomic DNA was extracted from peripheral blood leukocytes (3–5 ml) using an Agilent SureSelect Human All Exome V6 Kit (Agilent Technologies, Santa Clara, CA, USA), according to the manufacturer's instructions. After DNA extraction, the products were sequenced on a NovaSeq 6000 sequencer (Illumina, San Diego, CA, USA), with a reading length of 150 bp. The NovaSeq 6000 platform was used to examine >99% of the target regions. Sequence alignment was performed according to GRCh37/UCSC hg19. The variants were classified into five categories according to the American College of Medical Genetics and Genomics guidelines for the interpretation of genetic variants:^10^ pathogenic, likely pathogenic, uncertain significance, likely benign, and benign. The genetic variants were filtered using population databases such as the 1000 Genomes Project (1000G) (http://www.internationalgenome.org/), ESP6500, dbSNP, ClinVar, Exome Aggregation Consortium, and Human Gene Mutation Database. Furthermore, we studied evolutionary conservation. Sanger sequencing was used to validate suspected variants in the parents.

### 2.3 Bioinformatic analysis

The ClustalW program was used to perform sequence alignment of the protein of the *CWF19L1* gene (http://bioinfo.hku.hk/services/ashingt/cgi‐bin/clustalw_in.pl). Deleteriousness prediction of the variants of the gene was performed to evaluate their impact on protein function using SIFT (http://sift.bii.a‐star.edu.sg/), Polyphen2, Mutation Taster (http://www.mutationtaster.org/), CADD (https://cadd.gs.washington.edu/), and PROVEAN (Table 1). The three‐dimensional structure of the protein variants was determined using SWISS_MODEL (http://swissmodel.expasy.org/).

**TABLE 1:** Pathogenicity predictions of the two variants. D, damaging; A disease_causing_automatic.

Gene	Chromosome(hg19)	Nucleotide	Amino Acid	Crowd Frequency	SIFT	Polyphen2	MutationTaster	CADD	PROVEAN
*CWF19L1*	chr10:101993044	c.1557delGAG	p.Glu519del	(gnomAD v2.1.1) 0.0001	–	–	–	–	D
*CWF19L1*	chr10:101997963	c.1070G > T	p.Gly357Val	–	D(0.024)	D(0.997)	A(1)	D(32)	D(−5.82)

## 3 RESULTS

### 3.1 Clinical data

**FIGURE 2:**
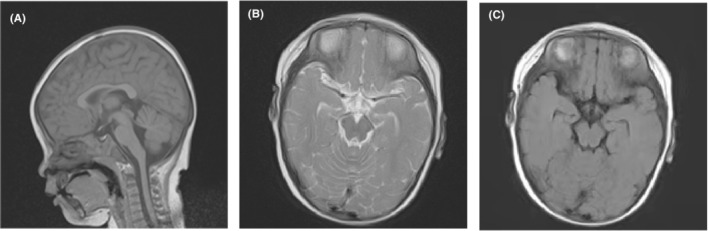
Magnetic resonance imaging of the brain of the proband showing normal findings without cerebellar or vermis atrophy. The sections are sagittal T1, axial T2, and axial fluid‐attenuated inversion recovery (from left to right).

Our patient was a Chinese boy born to non‐consanguineous parents at term after an uncomplicated delivery and with a birth weight of 3000 g. The boy sat without support at age 7 months and started to walk without support at 13 months. He was previously healthy and had normal growth and development until the age of 5 years. Initially, he was walking unsteadily with frequent falling, and his disease course gradually progressed over months to years, with subsequent involvement of speech and movements. He had unsteady, broad‐based gait, ataxic upper limb movements, and dysarthria. Furthermore, in addition to being a slow learner, he showed slurred speech, slowed performance, and difficulty in passing examinations. The intelligence quotient of our patient showed cognitive impairment, as reported by neuropsychological tests. Physical evaluation of the patient revealed a height of 120 cm (P25), a weight of 21 kg (P10), and a skull circumference of 52 cm (P50). His medical history was unremarkable, including exposure to drugs or toxins, systemic malignancy, trauma, and endocrinopathy. The findings of brain magnetic resonance imaging (MRI) at the age of 8 years were normal (Figure 2A–C). Therefore, ataxia was initially ruled out due to the absence of cerebellar atrophy on MRI. However, his gait was impaired with an inability to perform tandem gait, and a neurological examination revealed gait ataxia and finger‐to‐nose dysmetria. The patient had normal strength without dystonic movement disorders. His parents characterized him as friendly and sociable.

An extensive metabolic workup of the blood and urine did not reveal any abnormalities. Fundoscopic examination, electroencephalography, electromyography, and visual examination were normal at 8 years. He had no family history of any hereditary or neurological diseases.

### 3.2 Genetic analysis and co‐segregation in the family

**FIGURE 3:**
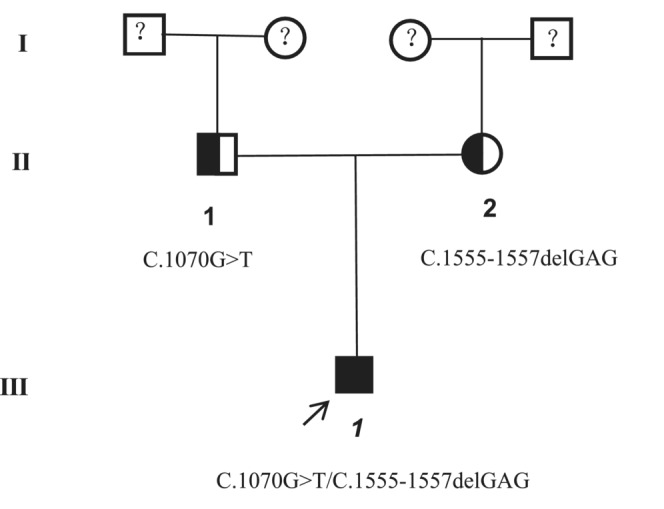
Pedigree of the Chinese family with autosomal recessive cerebellar ataxia. The proband is indicated in black. *CWF19L1* variants are indicated, illustrating that the c.1070G > T and c.1555_c.1557delGAG variants were inherited from the father and mother, respectively.

**FIGURE 4:**
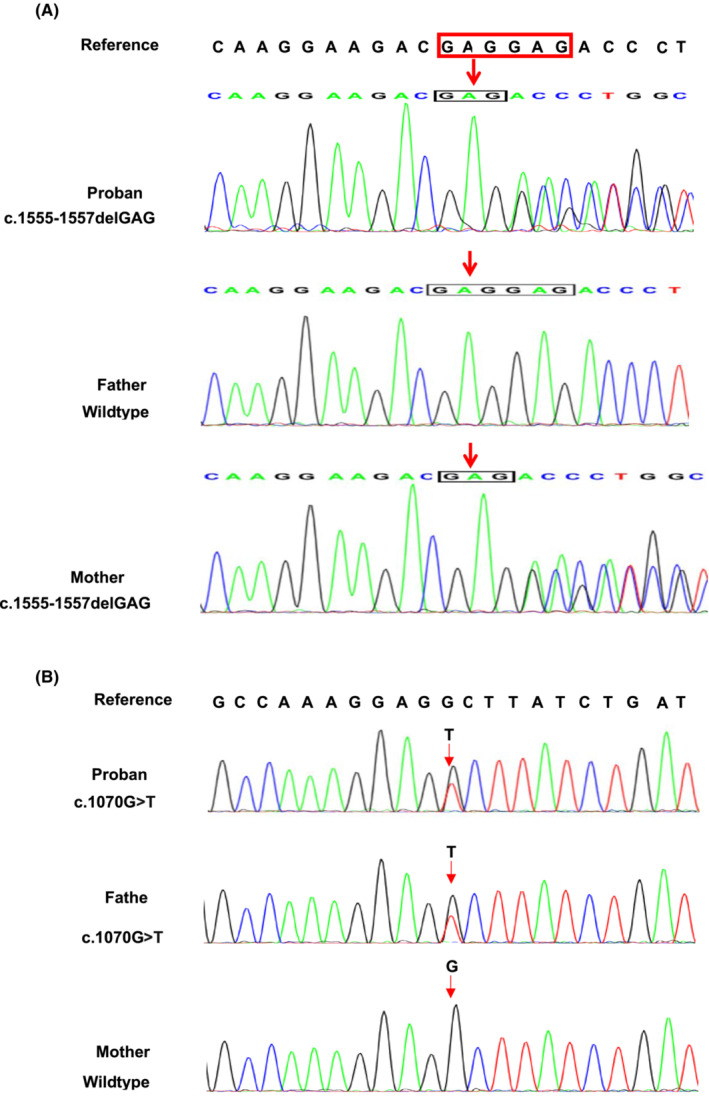
Sanger sequencing showing the *CWF19L1* c.1555_c.1557delGA (A) and *CWF19L1* c.1070G > T variants (B). Red arrows indicate the variant sites.

**FIGURE 5:**
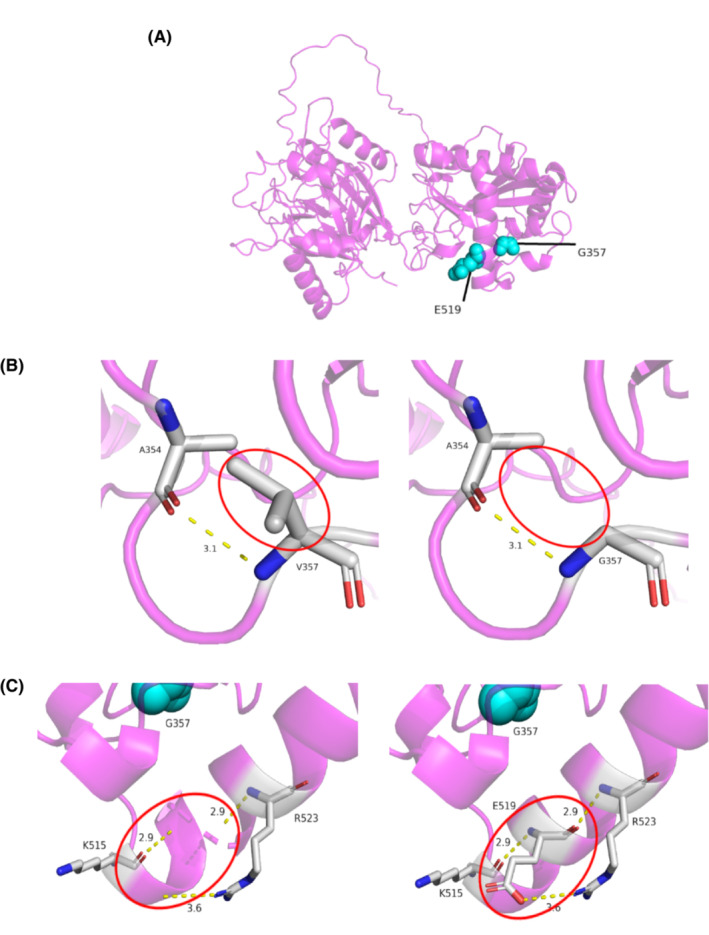
Modeled structure of the human C19L1 protein. (A) Structure of the wildtype C19L1 protein. G357 and E519 residues are annotated in green. (B) Amino acid and conformation changes in the wild‐type and mutant type p.Gly357Val polypeptide. (C) Amino acid and conformation changes in the wild‐type and mutant type p.Glu519del polypeptide.

Two novel compound heterozygous variants of *CWF19L1* (c.1555_c.1557delGAG in exon 14 and c.1070G > T in exon11) (NM_018294) were identified in the proband (III‐1) (Figure 3) using whole‐exome sequencing. Co‐segregation verification of these two variants was performed by Sanger sequencing (Figure 4). The variant c.1070G > T, inherited from the father (II‐1) (Figure 3), substitutes the polar amino acid glycine (Gly) with the nonpolar amino acid valine (Val) at position 357 (Figure 5B). The variant c.1555_c.1557delGAG inherited from the mother (II‐2) (Figure 3) led to the deletion of glutamic acid (Glu) at position 519 (Figure 5C). Therefore, the patient in our study was compound heterozygous. None of these variants have been reported previously. All variants are rare and have not been previously presented in 1000 Genomes Consortium Phase 3 (based on GRCh38) (http://www.ensembl.org/), Exome Variant Server database (http://evs.gs.washington.edu/EVS/), or dbSNP (https://www.ncbi.nlm.nih.gov/SNP/). Based on American College of Medical Genetics and Genomics guidelines, the variants detected in this case report were of unclear significance. Using professional functional study software, these variants were predicted to be potentially damaging. As previously reported, variants of this gene are known to cause autosomal recessive disorders such as spinocerebellar ataxia and autosomal recessive 17 (SCAR17) (OMIM #616127).

### 3.3 Structure–function correlations of *CWF19L1* variants

**FIGURE 6:**
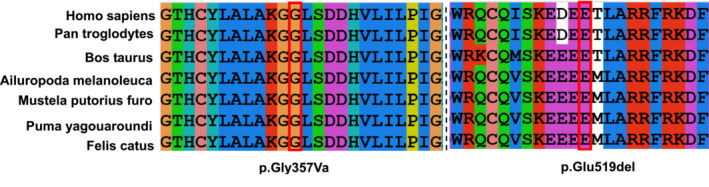
Conservation of the p.Gly357Val and p.Glu519del variants across various species.

SWISS‐MODEL was used to simulate the prominent amino acids and conformational changes in the polypeptide (Figure 5A). The protein domain of C19L1 was predicted to contain an MMP domain (blue) (Figure 1), HIT domain (green), and C‐terminal Cwf‐J‐C domain (red). Furthermore, Glu 519 and Gly 357 are conserved throughout the evolution of C19L1 in mammalian species (Figure 6). The missense variant p.Gly357Val is present in the HIT domain, leading to a change in the polar amino acid Gly to the nonpolar amino acid Val in the C19L1 protein, which would affect the curling of the structure of the C19L1 protein. Another variant (p. Glu519del) is contained in the C‐terminal Cwf‐J‐C domain and near the end of the C19L1 protein, leading to a deletion of the polar amino acid Gly, which might affect the spiral knot of the structure of the protein. These variants are highly conserved throughout evolution and might induce a conformational change that would substantially destroy the activity and function of the protein, suggesting that the variants could play a vital role in maintaining the stability and function of proteins.

## 4 DISCUSSION

*CWF19L1* has been reported to be associated with a similar human phenotype, such as cerebellar ataxia and intellectual disability.^9^ We found that compound heterozygous variants c.1555_c.1557delGAG and c.1070G > T in *CWF19L1* may be related to ARCA in the Chinese family evaluated in this case report. The clinical features of our patient were consistent with those from other reports.^1^, ^2^, ^3^, ^4^, ^5^ The variations of *CWF19L1* were assumed to be the genetic etiology of ARCA in this family case. The finding enriches the mutation spectrum of *CWF19L1* gene and laid a foundation for future studies on the correlation between genotype and phenotype.

In 2014, Burns et al^9^ reported the first variant of the *CWF19L1* gene identified in a Turkish family with ARCA. Globally, four previous publications have reported the clinical characteristics and genetic analysis of six patients with novel variants of *CWF19L1* (Table 2). Moreover, total five variants have been reported to be associated with ARCA, including two missense variants (p.D13H and p.D132G), one frame shift variant (p.[P156Hfs*33]), one nonsense variant (p.K316*), and one splice variant (c.964 + 1G > A). The p.G357V variant found in this case report was the third missense variant. To date, multiple studies have investigated the effect of the *CWF19L1* variant on the physical structure and function of the C19L1 protein.^9^, ^10^, ^11^ However, its molecular function remains unknown.^14^ Evers et al^11^ presented a frameshift *CWF19L1* variant c.467delC, indicating a loss‐of‐function mechanism, which introduces a premature stop codon at amino acid 189 p.(P156Hfs*33). A previous study by Nguyen et al.^10^ described two variants, one of which led to a premature stop codon (p. K316*) that resulted in nonsense‐mediated messenger RNA (mRNA) decay. The other variant (P.D13H) affects protein stability or function because it changes the negatively charged highly conservative aspartate into positively charged histidine. Meanwhile, Burns et al^9^ reported a variant in *CWF19L1* with an obligatory splice site that leads to a change in the first base of the intron 9 form G to A (c.964 + 1G > A), which causes exon skipping and decreased mRNA, leading to loss of function. Consistent with these reports, morpholino‐mediated knockdown of *CWF19L1* in zebrafish led to defective cerebellar structure and abnormal motor behavior. Recently, Algahtani et al^8^ described a missense variant, c.395A > G, that changes Asp to Gly and is associated with a milder presentation of the phenotype. Although the existing findings cannot sufficiently explain the molecular mechanism of ARCA, they could be used as scientific evidence for the diagnosis.

**TABLE 2:** Comparison of phenotypes of or patients with the six previously reported patients with pathogenic variants in *CWF19L1*

Age and sex (y)	Age of onset (y)	Ataxia	Dysarthria	Walking	DTR	Microcephaly	ID	MRI	Ophthalmological involvement	Seizures	Dystonia
Present											
8 M	5	Mild	+	13 mo	Brisk		+	Normal			
Evers et al^9^, ^11^											
9 M	1	Mild		13 mo	NR	+	+	Cerebellar atrophy (vermian > hemispheral)			
Nguyen et al^8^, ^10^											
10 F	5	Mild	+	18 mo	Normal		+	Cerebellar atrophy(vermian > hemispheral)	Oculomotor apraxia		+
Bruns et al^7^, ^9^											
12 M	Congenital	Mild	+	20 mo	Brisk						
6 F	Congenital	Moderate	+	18 mo	Brisk		+	Cerebellar atrophy(vermian>hemispheral)			
Hussein et al^6^, ^8^											
33 F	30	Moderate	+	NA	Brisk		+	Cerebellar atrophy(vermian>hemispheral)		+	
29 F	29	Mild	+	NA	Brisk		+	Cerebellar atrophy(vermian>hemispheral)		+	

Abbreviations: DTR, deep tendon reflex; F, female; ID, intellectual disability; MRI, magnetic resonance imaging; M, male; mo, month; NR, not reported; NA, not available; y, years.

Reviewing the literatures, most ARCAs have heterogeneous age at onset, severity of disease progression, and frequency of extra‐cerebellar and systemic signs.^15^, ^16^, ^17^, ^18^, ^19^ These novel variants are likely to reduce the severity of the phenotype compared with that in patients with homozygous or compound heterozygous nonsense variants. This conclusion provides a reasonable explanation for the clinical severity of this case. In this case report, the clinical presentation of ataxia started in childhood without cerebellar atrophy, suggesting a functional rather than a developmental role for this protein. More studies are required to determine the role of this protein in brain development and function. We believe that our patient had a milder phenotype of *CWF19L1*/SCAR17, as he could still walk, although unsteadily, with a normal brain MRI. Whether the milder phenotype is related to the variant type or location remains controversial and requires further investigation.

*CWF19L1*, expressed throughout the brain, encodes a group of Cwf19‐like proteins, which are postulated to play a role in cell cycle control and endosomal trafficking.^14^, ^20^, ^21^, ^22^ Cwf19 is a part of the Cdc5p complex involved in mRNA splicing and has nuclear localization with MMP domain.^23^, ^24^, ^25^, ^26^ The C‐terminal Cwf‐J domains of Cwf19‐like, which is involved in branched RNA metabolism, modulate the turnover of lariat intron pre‐mRNAs by the lariat‐debranching enzyme DBR1 and its homologs, suggesting a role in mRNA processing and its importance for proper brain function.^21^, ^27^, ^28^ Additionally, the three‐dimensional structure of *CWF19L1* protein was predicted, and it showed that the novel variant c.1557del leads to a loss of amino acid Glu 519, which changes the α‐helix and affects the curling of the structure of the C19L1 protein. The missense variant p.Gly357Val changes the polar amino acid Gly to the nonpolar amino acid Val in the C19L1 protein, which would influence the function and stability of the protein. Recently, zebrafish animal models have been widely used to study variants in ataxia genes.^29^, ^30^, ^31^ In previous studies,^29^, ^30^ knockdown of *CWF19L1* in fish caused abnormal motor behavior and alteration of cerebellar structure, showing that the loss of *CWF19L1* is associated with an abnormal motor phenotype. Hence, we assumed that the variants could be the genetic etiology of the ARCA in the family case. Nevertheless, the genetic effect of the variants needs to be verified in future study.

## 5 CONCLUSION

We speculate that *CWF19L1* is the causative gene of ARCA in this family. Our study showed that these single‐nucleotide polymorphisms co‐segregated with the ataxia phenotype. Nevertheless, functional validation using animal and cell experiments should be conducted in the future. A combination of clinical evaluation and genetic analysis is recommended for the diagnosis of ARCA subtypes. More studies are needed to elucidate the detailed clinical characteristics of CWF19L1‐dependent ARCA.

## FUNDING INFORMATION

This work was supported by the National Natural Science Foundation of China (82171701 and 82070834), the Medical Science and Technology Project of Zhejiang Province (2022YK839), the Social Programs of the Wenzhou Technology Bureau (2020Y0419), and the Zhejiang Medical Association (2020ZYC‐B23).

## CONFLICT OF INTEREST

The authors have no conflicts of interest to declare.

## Data Availability

Data supporting the findings of this study are available from the corresponding author upon reasonable request.
